# Mandibular Bone Loss after Masticatory Muscles Intervention with Botulinum Toxin: An Approach from Basic Research to Clinical Findings

**DOI:** 10.3390/toxins11020084

**Published:** 2019-02-01

**Authors:** Julián Balanta-Melo, Viviana Toro-Ibacache, Kornelius Kupczik, Sonja Buvinic

**Affiliations:** 1 Institute for Research in Dental Sciences, Faculty of Dentistry, Universidad de Chile, Santiago 8380492, Chile; julian.balanta@correounivalle.edu.co (J.B.-M.); mtoroibacache@odontologia.uchile.cl (V.T.-I.); 2 School of Dentistry, Universidad del Valle, Cali 760043, Colombia; 3 Max Planck Weizmann Center for Integrative Archaeology and Anthropology, Max Planck Institute for Evolutionary Anthropology, 04103 Leipzig, Germany; kornelius_kupczik@eva.mpg.de; 4 Center for Quantitative Analysis in Dental Anthropology, Faculty of Dentistry, Universidad de Chile, Santiago 8380492, Chile; 5 Department of Human Evolution, Max Planck Institute for Evolutionary Anthropology, 04103 Leipzig, Germany; 6 Center for Exercise, Metabolism and Cancer Studies CEMC2016, Faculty of Medicine, Universidad de Chile, Independencia 8380453, Chile

**Keywords:** botulinum toxin type A, bone quality, muscle atrophy, temporomandibular joint, mandibular condyle, alveolar process, alveolar bone loss

## Abstract

The injection of botulinum toxin type A (BoNT/A) in the masticatory muscles, to cause its temporary paralysis, is a widely used intervention for clinical disorders such as oromandibular dystonia, sleep bruxism, and aesthetics (i.e., masseteric hypertrophy). Considering that muscle contraction is required for mechano-transduction to maintain bone homeostasis, it is relevant to address the bone adverse effects associated with muscle condition after this intervention. Our aim is to condense the current and relevant literature about mandibular bone loss in fully mature mammals after BoNT/A intervention in the masticatory muscles. Here, we compile evidence from animal models (mice, rats, and rabbits) to clinical studies, demonstrating that BoNT/A-induced masticatory muscle atrophy promotes mandibular bone loss. Mandibular bone-related adverse effects involve cellular and metabolic changes, microstructure degradation, and morphological alterations. While bone loss has been detected at the mandibular condyle or alveolar bone, cellular and molecular mechanisms involved in this process must still be elucidated. Further basic research could provide evidence for designing strategies to control the undesired effects on bone during the therapeutic use of BoNT/A. However, in the meantime, we consider it essential that patients treated with BoNT/A in the masticatory muscles be warned about a putative collateral mandibular bone damage.

## 1. Introduction

The intervention of the masticatory muscles with botulinum toxin is increasing among general and specialized dentists, aiming to reduce the activity of muscles such as the masseter during parafunctions like sleep bruxism and oral movement disorders (i.e., oromandibular dystonia) [1,2,3]. In addition, the transitory paralysis caused by this neurotoxin results in masseter atrophy, which is intended for aesthetic alterations like benign masseteric hypertrophy [1,4]. However, the lack of evidence regarding its effectiveness and the potential adverse effects in associated musculoskeletal structures have raised interest in scientifically assessing the latter, something that has not been considered by many clinical practitioners until now [2,3,5,6,7,8].

The mammalian masticatory apparatus is a complex and specialized anatomical system necessary for functions such as mastication, self-defense, and social relationships among others. Its main components are the teeth, the maxillary and mandibular bones, the temporomandibular joint (TMJ), and the masticatory muscles, with a common origin in the first and second pharyngeal arches [9,10,11]. The masticatory muscles (medial and lateral pterygoids, temporalis, and masseter) work synchronously to provide the mandibular movements, with the TMJ as a fulcrum. The TMJ articulates the mandibular condyle with the mandibular fossa in the skull base. The mandibular condyle exhibits an articular surface covered with fibrocartilage and a subjacent subchondral bone [12,13,14]. It is also a growth center that maintains its activity in adult individuals, which allows it to adapt to external stimuli changes [15].

During ontogeny, the structure and function of masticatory muscles are necessary for the proper development of the mammalian TMJ, as well as mastication performance [13,16]. Moreover, the biomechanical input from the masticatory muscles is required during adulthood to maintain the homeostasis of the joint [17,18]. The functional and/or structural alterations in one or more of the components of the TMJ are recognized as temporomandibular disorders (TMDs), grouped by muscular, articular or developmental conditions [19,20]. The TMDs include several pathological conditions that severely impair the quality of life, with a high cost in both diagnosis and management [19]. In addition, TMDs-related alterations affect, worldwide, the human daily activities such as talking, eating, and sleeping, with a higher prevalence in adult women between 20 and 40 years [21,22].

The mandible is an irregular bone that provides support and protection of the soft craniofacial tissues [23]; its alveolar bone houses the mandibular teeth [24], it allows for the insertion of masticatory muscles [10,25], while keeping general properties of bone as mineral reservoir [26] and primary center for hematopoiesis [27]. Since muscle function and bone homeostasis are related at biomechanical and biochemical levels [28], it remains necessary to understand the effect of masticatory muscle alterations, such as paralysis induced by botulinum toxin type A (BoNT/A) for therapeutic purposes [1,3,4,29], on the bone remodeling process in the mammalian mandible.

### 1.1. Bone Remodeling as an Integration Mechanism in the Masticatory Apparatus

In general, bone tissue is highly organized at several levels (from nano to macrostructure). It is composed of cortical bone with high mineral content and concentric laminae, and less mineralized and irregular trabecular bone [30,31,32]. Additionally, bone is highly innervated and vascularized, and presents a specialized cover called periosteum on the outside surface [33], with an internal analog called endosteum, in direct contact with the bone marrow [27,34].

Bone remodeling is the process of bone turnover after maturation, and the first process is the degradation of damaged tissue (bone resorption) [35,36]. The osteoclasts are a specialized cell line that performs the bone resorption process [37,38]. These cells result from the fusion of monocytes, recruited by specific molecular signals like the receptor activator of nuclear factor κ B ligand (RANKL), necessary for osteoclastogenesis [39,40]. After bone resorption, bone apposition is performed by the osteoblasts [41]. Once bone apposition is complete, a portion of the osteoblast population is covered by mineralized bone tissue and become differentiated into osteocytes [42,43]. The osteocytes represent almost 95% of the bone cells and they exhibit extended longevity, are sensitive to mechanical stimuli, and are the main source of RANKL in the trabecular bone [44]. The balance between RANKL and its soluble antagonist, Osteoprotegerin (OPG), determines the bone homeostasis and bone remodeling processes [45,46]. The reduction of bone mineral density (BMD), which reflects the bone’s mineral content, is called osteopenia. It precedes osteoporosis, which compromises trabecular bone microstructure and quality under different conditions such as immobilization, lack of gravity, hormonal alterations, among others [45]. Since most of the mandible is formed by bone, the adaptive capacity of this mineralized tissue is necessary for the integrity of the mammalian masticatory apparatus [47,48].

### 1.2. Mechanism of Action and Treatment of Oral Muscular Disorders: The Use of Botulinum Toxin Type A in Dentistry

Botulinum toxin is a neurotoxin produced by the anaerobic bacteria *Clostridium botulinum*. The most potent serotype is the A (BoNT/A) and it is composed of two chains; a heavy chain of 100 kDa and a light chain of 50 kDa [1,49,50]. The former acts as a specific ligand for presynaptic membrane receptors in cholinergic nerve endings (such as NMJ) and the latter, once inside the motor neuron, cleaves the SNAP25 protein through zinc-dependent proteolytic activity; SNAP25 is part of the SNARE complex, which is necessary for exocytosis [49]. Therefore, acetylcholine release to the NMJ cleft is blocked, and skeletal muscle is temporally paralyzed and subsequently atrophied [49,50,51]. This is an expected outcome in dentistry for oral muscular disorders such as sleep bruxism [3] or aesthetic conditions like masseteric hypertrophy [1,4]. In addition, the block of neurotransmitter release (like Substance P) also seems to be helpful for the treatment of myofascial pain [1,29]. However, there is a lack of high-quality evidence that supports the effectiveness of this intervention for such mentioned disorders. Moreover, this neurotoxin is not approved for interventions in the masticatory apparatus, according to the US Food and Drug Administration (FDA) [52,53]; as such, its clinical use is off-label. Despite this, BoNT/A has been considered as an option for some clinical trials [54] and is commonly used by dental practitioners and aesthetic therapists. This raises the concern about the safety of this procedure [5,6,8].

### 1.3. BoNT/A and Bone Loss at the Masticatory Apparatus

Considering that muscle contraction drives the mechano-transduction and molecular signaling required for bone homeostasis, what happens to mandibular bone when masticatory muscles are paralyzed? Pre-clinical evidence showed that masticatory muscle atrophy induced by BoNT/A impairs craniofacial bone development by reducing the size of particular regions of mandible (such as mandibular condyle) and altering its morphology, when compared with normally developed individuals [55,56,57,58,59,60,61]. However, the adverse effects of this intervention in adult individuals remains poorly understood. Therefore, the aim of this work is to condense the current and relevant literature about the mandibular bone loss in fully mature mammals after BoNT/A intervention in the masticatory muscles.

## 2. Methods

An electronic literature search of the databases PubMed, EMBASE and Scopus, was conducted in January 2019 by two independent reviewers with the aim to identify the relevant literature regarding the effect of masticatory muscle atrophy induced by botulinum toxin type A (BoNT/A) on the mandibular bone structure of adult individuals. No time range was considered, and the search was restricted to English language. The following search strategy combining Medical Subject Headings (MeSH), search terms, and truncated terms was used:

((((((((((("Mandible"[Mesh]) OR Mandib*[Title/Abstract]) OR "Temporomandibular Joint"[Mesh]) OR "Mandibular Condyle"[Mesh]) OR Mandibular head*[Title/Abstract]) OR Mandibular condyle*[Title/Abstract]) OR Subchondral bone*[Title/Abstract]) OR "Alveolar Bone Loss"[Mesh]) OR "Alveolar Process"[Mesh]) OR Alveolar bone*[Title/Abstract])) AND ((("Botulinum Toxins, Type A"[Mesh]) OR "Botulinum Toxins"[Mesh]) OR Botulinum toxin*[Title/Abstract])

Additionally, a Google Scholar search was performed to identify any other relevant studies. The search terms were screened initially in the title and the abstract before selection for full-text review. Only publications that report mandibular bone effects after BoNT/A intervention in the masticatory muscles without any other conditions (e.g., induced osteoporosis) were considered. Previously published narrative or systematic reviews and conference abstracts were considered as exclusion criteria. Bone parameters such as histomorphometry and microtomography were considered as primary outcomes; time of evaluation after intervention, molecular expression (gene and/or protein), and Bone Mineral Density (BMD) from affected bones were considered as secondary outcomes.

## 3. Results

**Figure 1:**
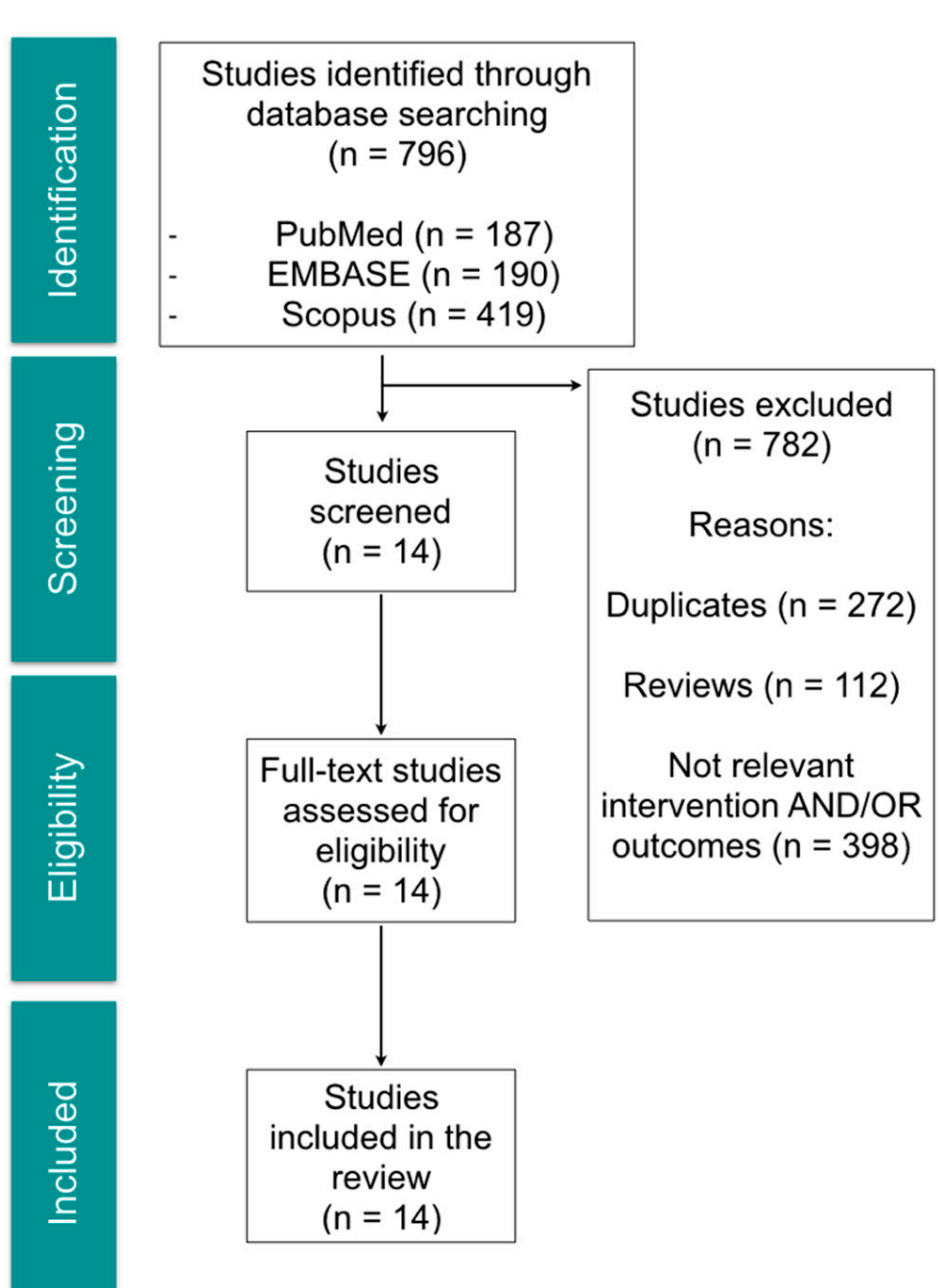
Flow chart of selection process for relevant studies.

The search strategy retrieved a total of 796 articles. After title and abstract evaluation, 14 articles were included for full-text review. From the selected articles, 10 were experimental studies using animal models (mice, rats and rabbits) and 4 were reports from human studies: one clinical case-report, two retrospective studies and one clinical trial. The selection process is shown in Figure 1, and the descriptive summary of the articles that met the inclusion criteria in Table 1.

Our literature search retrieved preclinical and clinical studies related to mandibular bone changes after BoNT/A intervention in the masticatory muscles. Taken together, it is possible to describe the main results based on three different levels for bone quality evaluation [73]: cellular and metabolic changes, microstructural changes and morphological changes.

### 3.1. Cellular and Metabolic Changes

**Figure 2:**
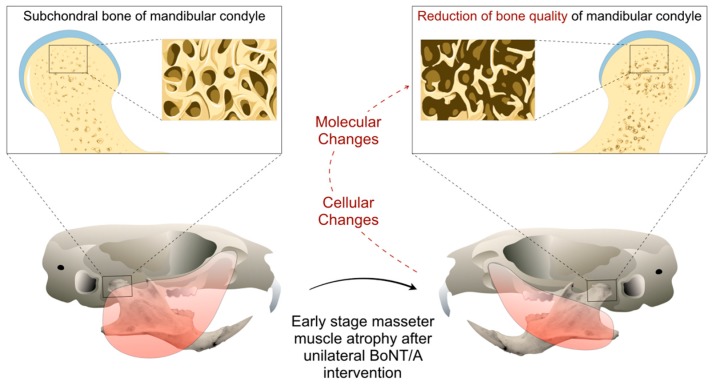
Mouse model of mandibular condyle degradation during the early stage (2 weeks) of BoNT/A-induced masseter muscle atrophy in adult animals.

**Figure 3:**
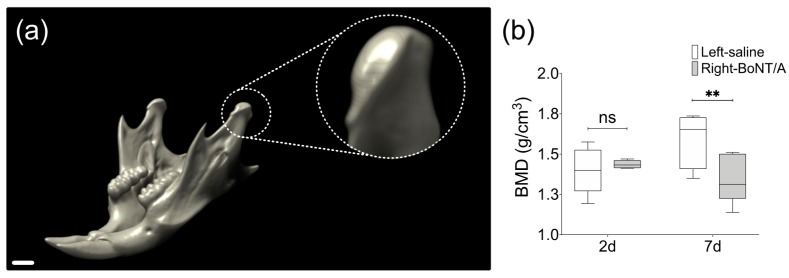
Bone Mineral Density (BMD) from mandibular condyles of adult male mice 2 days and 7 days after unilateral BoNT/A intervention in the right masseter muscle. (**a**) 3D view of mouse mandible performed with DataViewer (v1.5.6.2, Bruker microCT). The scan was carried out under the following parameters: SkyScan 1278 (Bruker), Voltage 65 kV, Current 692 µA, Aluminum filter 1 mm, voxel size 51.48 µm and reconstruction program NRecon (v1.7.4.2, Bruker microCT). Dotted circle: Close up of volume of interest, the mandibular condyle. Scale bar: 1 mm. (**b**) Measurement of BMD in samples from both sides of experimental individuals 2 days and 7 days after BoNT/A injection, obtained with the CT Analyzer (v1.18.4.0, Bruker microCT). Min to Max; n = 5 per day; paired *t*-test between samples from the same individual; Shapiro Wilk test: *p* > 0.05; ** *p* < 0.01; ns: non-significant.

Tsai et al. (2010) found no differences in BMD from the whole mandible samples in a rat model of unilateral injection of BoNT/A in the masseter muscle, three months after intervention [72]. However, a 3–4% reduction in BMD was described in mandibular condyles of unilateral BoNT/A-injected young female mice four weeks after intervention, comparing the injected side with the contralateral non-injected side or with samples from animals without intervention [63,66]. Unpublished results from our mouse model (Figure 2) show even earlier changes in BMD [74] (Figure 3). In adult mice, seven days after unilateral BoNT/A intervention in the masseter muscle, a significant reduction in the BMD of the mandibular condyles from the BoNT/A-injected side, compared to the saline-injected side was found (Figure 3).

A significant reduction in bone remodeling processes of the samples from the experimental side was detected in young adult female mice as a decrease in both osteoclast activity (measured with TRAP staining) [63,66] and bone mineralization (assessed through alkaline phosphatase staining and fluorescent dyes for mineralized bone) after four weeks [63]. In contrast to these findings, Shi et al. reported, in adult female rats, a significant increase of the TRAP staining in the subchondral bone of mandibular condyles four weeks after bilateral injection of BoNT/A in the masseter muscles, when compared with samples from animals bilaterally injected with saline solution [64]. However, different techniques were implemented to assess TRAP staining: In the samples from mice [63,66], a fluorescent approach was employed using TRAP-positive pixels for quantification, whereas the study with rats used an immunohistochemical procedure with multinucleated positive TRAP cells quantitation [64]. In addition, in a pilot study with adult male mice, we demonstrated a significant increase in mRNA levels of the bone resorption marker RANKL in extracts from mandibular condyles just two days after unilateral BoNT/A injection in the masseter muscle [51].

Another result that involves a cellular change in the bone tissue was reported by Dutra et al. [66]. They founded an increased number of apoptotic cells (visualized by TUNEL reaction) in the subchondral bone of the mandibular condyle from the BoNT/A-injected side in young adult female mice, four weeks after intervention [66]. However, there was no identification of the cell type labeled for apoptosis.

### 3.2. Microstructural Changes

In a pilot study, we implemented a mouse model to determine the effect of unilateral injection of BoNT/A in the masseter muscle on mandibular condyle microstructure in adult male mice [51]. We found a significant reduction in bone per tissue area (B.Ar/T.Ar; 30%) and trabecular thickness (Tb.Th; 55%) of the subchondral bone in the treated side, assessed by histomorphometry of representative slices from the middle portion of the mandibular condyles two weeks after intervention [51]. In adult male rats, the same intervention showed a significant decrease in the cortical thickness and the trabecular bone in coronal slices at coronoid and molar levels, three months after following intervention [72]. Additionally, using 2D bone histomorphometry in adult female rabbits, a similar significant reduction in the subchondral bone (20%) was found four weeks after unilateral intervention in the masseter muscle, with a statistically non-significant recovery at 12 weeks, when compared with the control side injected with saline solution [67]. Moreover, a microCT evaluation of 2D representative slices from the middle portion of the mandibular condyle in rabbits showed a significant reduction of B.Ar/T.Ar four weeks after intervention (compared with the control side); the difference in this bone parameter was still statistically significant at 12 weeks [70]. In addition, a loss of alveolar bone (at molar level) was detected in the experimental side, but no longer detected 12 weeks after BoNT/A intervention [70]. In adult male rats, the unilateral injection of BoNT/A in the masseter and the temporalis muscles resulted in a significant reduction of B.Ar/T.Ar in the alveolar bone and the mandibular condyle (20 and 35%, respectively) when compared with the control side four weeks after, using microCT imaging [68].

**Figure 4:**
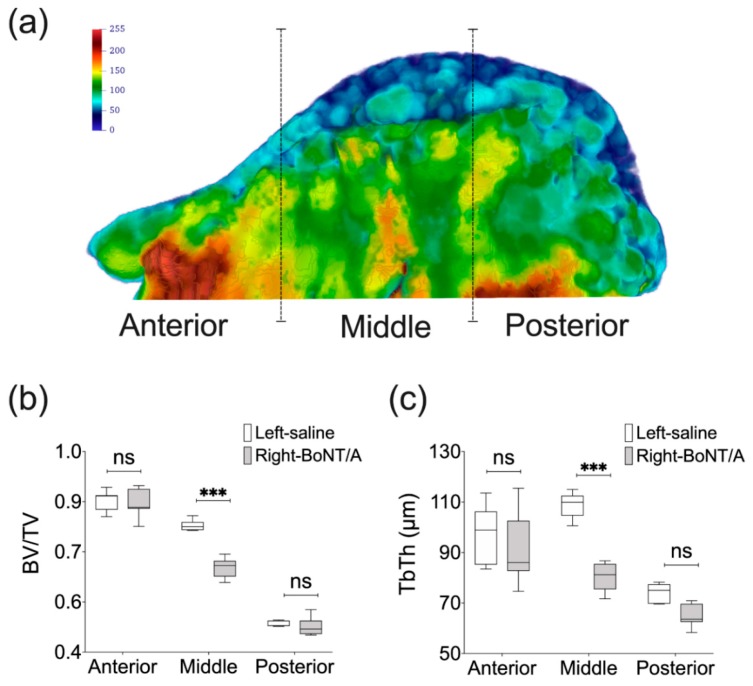
MicroCT-based histomorphometry of the mandibular condyle in adult male mice. (**a**) Parasagittal view of 3D Trabecular Thickness (Tb.Th) depiction of a representative mandibular condyle from BoNT/A-injected side in adult male mice, 2 weeks after (performed with Paraview, v5.4.1). Samples are from three locations and contain the same number of microCT slices. Color scale in gray values. (**b**) A significant reduction of Bone Volume Fraction (BV/TV) was detected only for the middle portion of the mandibular condyles from BoNT/A-injected sides, when compared with saline-injected control. A higher reduction was found when compared with the whole volume assessment of the mandibular condyle (16 vs 11%). (**c**) The same result was found for the Tb.Th, with a significant difference in the middle portion of the mandibular condyle, and a higher difference between sides (26%) (Min to Max; n = 7; One-way ANOVA, *p*-values after Bonferroni´s multiple comparisons test; *** *p* < 0.001; ns: non-significant).

In comparison with 2D evaluations, the 3D (volumetric) analyses of mandibles using microCT technology offers a more complete picture of how bone loss presents following BoNT/A intervention. It has been demonstrated for example that the extent of bone loss is less than expected from 2D analyses, albeit still significant. An advanced 3D analysis using high resolution microCT showed a reduction of 10–11% in bone volume fraction (BV/TV) in the mandibular condyle of young adult female mice [63] and adult male mice [62] four weeks and two weeks after, respectively. A higher value was reported in another study (loss of 21% in BV/TV), but these mice were younger (five weeks old) [66]. In mandibular condyles of adult male mice, Tb.Th was also significantly reduced, while trabecular number (Tb.N) and trabecular density (Conn.D) were significantly increased at two weeks. However, no statistically difference was detected for these bone parameters in the alveolar bone at the first molar level [62]. On the other hand, in the mandibular condyles of young adult female mice, Tb.Th was significant reduced (17%) and trabecular separation (Tb.Sp) was increased by 18% at four weeks, compared with control side [66]. Moreover, unpublished results from our lab using a high resolution microCT approach previously described [62] has confirmed that the significant reduction of the bone microstructure parameters such as BV/TV and Tb.Th is more pronounced in the middle portion of the mandibular condyle [74] (Figure 4). This may explain why findings of studies that only assessed the middle portion of the mandibular condyle using a 2D approach reported higher bone loss than those using 3D evaluation. Interestingly, bilateral injection of BoNT/A in the masseter muscles may have a greater adverse effect on mandibular condyle microstructure. As shown by Shi et al. in adult female rats, at 4 weeks, BV/TV and Tb.Th of the mandibular condyles were both decreased by a 50% and the Tb.Sp was significantly increased when compared with samples from control individuals [64].

In humans, a pilot study in adult women suggested a potential damage of the mandibular condyle after BoNT/A intervention in the masticatory muscles [69]. Patients were administered between 2 and 7 BoNT/A injections with an average time of three months between sessions. Imaging analysis from two independent radiologists using Cone Beam Computerized Tomography (CBCT) detected significant reduction of trabecular bone density and cortical thickness, when compared with a similar cohort of unexposed individuals [69]. With the same CBCT approach, a clinical trial in patients with masseteric hypertrophy (adult men and women) showed a significant reduction of the bone volume in the mandibular angle after two different sessions of bilateral BoNT/A injections in the masseter muscles, with a time of four months between each one, and assessment six months after the first BoNT/A intervention [8]. In contrast, a retrospective study in adult women with squared-face as chief complaint, found no significant difference in the whole mandible volume and the cortical thickness of the mandibular ramus three months after of bilateral BoNT/A injections in the masseter muscles [71].

### 3.3. Morphological Changes

Tsai et al. reported several mandibular changes three months after unilateral BoNT/A intervention in the masseter muscle of adult male rats [72]. Linear measurements demonstrated a significant reduction of the mandibular ramus, and a significant increase in the length of the mandible, measured between the mandibular condyle and the tip of the mandibular incisor [72]. Additionally, they described qualitative morphological alterations in the insertion of the masticatory muscles. This is consistent with our findings in the insertion site of the BoNT/A-injected masseter muscle on the vestibular side of the mandible, close to the first molar zone [62]. However, assessment of bone microstructure in this specific portion of the alveolar process revealed no significant differences when compared with saline-injected control side, 2 weeks after [62]. The 3D assessment of condyle shape using geometric morphometrics showed that 2 weeks after unilateral BoNT/A intervention in the masseter muscle, mouse mandibular condyles of injected side were more extended anteroposteriorly, with a decreased width, and exhibited a concave anterior-superior surface, when compared with samples from control side [62]. These findings contrasted with a study using young adult female mice, where a reduction in the anterior-posterior dimension of the mandibular condyle was found four weeks after unilateral BoNT/A intervention but without any alteration of mandibular length (between condyle and incisor) [66]. In humans, a clinical case-report of an adult woman that received repetitive BoNT/A injections in the masseter muscle every three months for the treatment of oromandibular dystonia showed a condylar bone resorption only in the BoNT/A-injected side, a morphological change detected with Dynamic Magnetic Resonance Imaging [5].

## 4. Discussion

The BoNT/A intervention is a promising tool in dentistry for the management of several clinical conditions, including those related to myofascial pain [1,7,29]. Moreover, for dentists, injecting BoNT/A in the facial region is not technically difficult, making this procedure highly available for patients in dentistry [1]. However, concerns about its effectiveness are based on clinical studies with a poor design and high risk of bias [2,3,7]. In addition, there are no indications for its use in the masticatory apparatus, and the adverse effects are rarely or never reported in the clinical trials, assuming the absence of effects beyond the injected muscles. Most of the relevant literature found here about mandibular damage after BoNT/A intervention in the masticatory apparatus comes from pre-clinical studies. However, heterogeneity in the design (i.e., animals used, brand of BoNT/A, dose equivalence, dose assessment methods, among others) does not make it possible to properly compare the results. Interestingly, the use of 3D technologies such as microCT adds significant improvements in the evaluation of bone effects in irregular structures like the mandibular condyle.

In clinical dentistry, the BoNT/A intervention in the masticatory muscles has been used for the treatment of several oral movement disorders such as oromandibular dystonia [75] and sleep bruxism [3] and aesthetics conditions like masseteric hypertrophy [1]. However, there is no official approval by the FDA for this therapeutic strategy [53]. In addition, safety considerations regarding the adverse effects of BoNT/A-induced masticatory muscle atrophy on mandibular bone from pre-clinical studies are relevant in order to avoid unnecessary risks in human trials. Thus, bone mandibular assessment during clinical trials should be considered as an important outcome after BoNT/A intervention in the masticatory muscles.

The muscle-bone crosstalk in the masticatory apparatus is still poorly understood, and results from animal experiments could be useful to study the cellular and molecular dynamics behind soft and hard tissue homeostasis. The establishment of a mouse model (Figure 2) allows us to design better and controlled experiments with less resources (compared with the use of larger animals), less time for intervention/response and easier genetic manipulation. However, it is necessary to consider the genetic and physiological differences between mice and humans before suggesting potential similarities in the bone tissue response during altered masticatory function induced by BoNT/A [76,77,78,79]. The bone remodeling process is quite a lot faster in mice (two weeks) when compared with humans (up to nine months) [76]. In addition, the bone loss after BoNT/A intervention in the skeletal muscle is strain-dependent in mice, which suggests that careful attention must be dedicated during experimental design and comparison between studies [80]. Interestingly, the molecular response during bone loss such as the increase of RANKL has been explored using genetic modified mice and represents some common features of the bone biology between these species with humans [81]. Therefore, although the mastication biomechanics in mice and animals is different, it is possible that they share cellular and molecular mechanisms related to bone remodeling processes. Without doubt it would be relevant to be able to evaluate these processes in humans. However, due to the bone damage observed by BoNT/A intervention of masseter muscles in the animal model, we do not consider it pertinent or ethical to conduct a clinical study.

Most of the studies that use rodents as models to evaluate the effects of BoNT/A-induced masticatory imbalance are highly focused on the mandibular condylar cartilage (MCC). This is important to highlight, because early effects of masseter muscle atrophy after BoNT/A intervention seem to be related to cellular/molecular and microstructural changes in the subchondral bone, preceding detectable damage of the MCC. Therefore, mouse models are important to understand the potential adverse effects of BoNT/A in the homeostasis of mandibular, as well as to unveil the cellular and molecular mechanisms responsible for these outcomes. Taken together, results from pre-clinical models using BoNT/A intervention could also shed light on understanding pathologies such as temporomandibular joint osteoarthritis, where changes in the remodeling process of subchondral bone have been suggested as an early manifestation of the disease [82].

Gathering evidence, we have found that the best way to calculate the dose of BoNT/A to be injected into the masseter muscle of different preclinical models is the relationship between the toxin units and the muscle mass. In this way, we have found that Botox® injection of 1.2–3.3 U/g masseter muscle is safe for interventions in mouse, rat and rabbit models (Table 2). This is an important parameter to consider when starting any pilot study, as it will reduce the loss of animals used in the calibration of the procedure. In addition, it is relevant to note that units of different brands of commercialized BoNT/A are not equivalent [52]; thus, it is highly necessary to consider the BoNT/A source when comparing evidence. The onabotulinumtoxin A molecule is the most reported BoNT/A in experimental studies in animals and it was reported in one clinical trial [8], whereas the abobotulinumtoxin A was used in another study in humans [71] (Table 2). However, all preclinical studies with bone-related outcomes used BoNT/A from the same company (Allergan, Inc.) [51,62,63,64,65,66,67,68,70,72], while both clinical studies used BoNT/A from Medytox, Inc. [8] and Ipsen Biopharm Ltd [71] (Table 2). Therefore, the brand and dosage of BoNT/A should be carefully considered when reviewing literature and evaluating the effectiveness or adverse effects of the procedures with BoNT/A.

## 5. Conclusions

The findings from pre-clinical studies reviewed here suggest that intervention with BoNT/A in the masticatory muscles presents adverse effects related to bone loss in the mandible at specific and time-dependent regions, such as the mandibular condyle and the alveolar process. However, the cellular and molecular mechanisms behind these phenomena remain to be fully understood. It will be relevant to address in the future if muscle atrophy/paralysis leads to osteopenia events through mechanical unloading, a deregulation of biochemical factors normally secreted by muscles to maintain bone homeostasis, or both. Further basic research may also provide tools for new indications and to control undesired effects. On the other hand, studies in humans are scarce and reported contrasting designs and results regarding mandibular bone loss effects. However, the only clinical trial reviewed here demonstrated mandibular angle bone loss after repetitive injections of BoNT/A in the masseter muscle. Therefore, the current reviewed evidence warnings about potential mandibular bone loss that may affect the temporomandibular joint and the alveolar bone around teeth, and this statement should be communicated to the patients before BoNT/A intervention in the masticatory muscles.

## Figures and Tables

**Table 1:** Summary of selected articles.

Author	Individuals	Intervention	Time after Intervention and Bone Evaluation Methods
Balanta-Melo et al. 2018 [62]	Adult male BALB/c mice (8–9 weeks-old)	Experimental group: 0.2 U BoNT/A in the right masseter and saline solution in the left masseter Control group: without intervention	2 weeks; 3D bone parameters from mandibular condyle and alveolar process, and shape analysis of mandibular condyle using microCT
Dutra et al. 2018 [63]	Young adult female C57BL/6J mice (6 weeks-old)	Experimental group: 0.3 U BoNT/A in the right masseter and no intervention in the left masseter Control group: without intervention	4 weeks; 3D bone parameters using microCT, BMD and histomorphometry from mandibular condyle
Shi et al. 2018 [64]	Young adult female Sprague-Dawley rats (5 weeks-old)	Experimental group: 2 U BoNT/A bilateral in both masseter muscles Control group: without intervention	4 weeks; 3D bone parameters using microCT and histomorphometry from mandibular condyle
Balanta-Melo et al. 2018 [51]	Adult male BALB/c mice (8 weeks-old)	Experimental group: 0.2 U BoNT/A in the right masseter and saline solution in the left masseter	2 weeks; bone histomorphometry and mRNA quantification from mandibular condyle
Aziz et al. 2017 [5]	Adult woman (55 years-old)	140 U BoNT/A quarterly in the left masseter	Morphology of the mandibular condyle (qualitative description) using diagnostic imaging (Dynamic Magnetic Resonance Imaging)
Lee et al. 2017 [8]	Adult men and women (28–48 years-old)	Experimental group I: 25 U BoNT/A bilaterally in the masseter muscles Experimental group II: 25 U BoNT/A bilaterally in the masseter muscles; repetition 4 months after the first intervention	6 months after first intervention; evaluation of bone volume in the mandibular angle using CBCT
Kün-Darbois et al. 2017 [65]	Adult male Sprague–Dawley rats (18 weeks-old)	Experimental group: 1 U BoNT/A unilaterally in the masseter and temporalis muscles Control group: unilateral injection of saline solution in the masseter and temporalis muscles	4 weeks; 3D bone parameters using microCT of mandibular condyles
Dutra et al. 2016 [66]	Young adult female transgenic mice (Col10a1) on a CD-1 background (5 weeks-old)	Experimental group: 0.3 U BoNT/A in the right masseter and no intervention in the left masseter	4 weeks; 3D bone parameters using microCT, BMD and histomorphometry of mandibular condyles
Matthys et al. 2015 [67]	Adult New Zeland white female rabbits (5 months-old)	Experimental group: 10 U BoNT/A unilateral in the masseter muscle Control group: unilateral injection of saline solution in the masseter muscle	4 weeks and 12 weeks; bone histomorphometry of mandibular condyles
Kün-Darbois et al. 2015 [68]	Adult male Sprague–Dawley rats (18 weeks-old)	Experimental group: 1 U BoNT/A unilateral in the masseter and the temporalis muscles Control group: unilateral injection of saline solution in the masseter and the temporalis muscles	4 weeks; 2D analysis of microCT slices from mandibular condyles and alveolar bone
Raphael et al. 2014 [69]	Adult women (Mean age 45 years-old)	Exposed group: Adult women with myofascial pain exposed to BoNT/A for treatment. No dose of BoNT/A reported. Unexposed group: Adult women with myofascial pain with no previous exposure to BoNT/A	CBCT 6-10 weeks after exposure to BoNT/A intervention
Rafferty et al. 2012 [70]	Adult New Zeland white female rabbits (5 months-old)	Experimental group: 10 U BoNT/A unilateral in the masseter muscle Control group: unilateral injection of saline solution in the masseter muscle	4 weeks and 12 weeks; 2D and 3D evaluation using microCT of mandibular condyles and alveolar bone
Chang et al. 2011 [71]	Adult women	Bilateral injection of 120 U BoNT/A in both masseter muscles	3 months; 3D analysis of cortical thickness of the mandibular ramus using CT
Tsai et al. 2010 [72]	Adult male Sprague-Dawley rats (8 weeks-old)	Experimental group: 7.5 U BoNT/A in the left masseter and saline solution in the right masseter	3 months; Linear measurements and BMD of mandibles 2D histomorphometry of slices at first molar and coronoid levels

BoNT/A, Botulinum toxin type A; BMD, Bone Mineral Density; U, Mouse Units; CT, computerized tomography; CBCT, cone-beam computerized tomography; 2D, two dimensional; and 3D, three dimensional.

**Table 2:** Summary of the characteristics of BoNT/A interventions in the included literature.

Individual	Average Masseter Mass (g)	Generic Name/Brand	Dose/Volume (U/ml)	Average BoNT/A Dose Per Masseter Mass (U/g)
Mouse [51,62,63,66]	0.075 [51]	*Onabotulinumtoxin A*; Botox®, Allergan Chile, Santiago, Chile [51,62] *Onabotulinumtoxin A*; Botox®, Allergan, Plc, Parsippany-Troy Hills, NJ, USA [63,66]	0.2–0.3/0.01–0.03	3.3
Rat [64,65,68,72]	1.1 [64]	*Onabotulinumtoxin A*; Botox®, Allergan Inc., Irvine, CA, USA [64,65,68] *Onabotulinumtoxin A*; Botox®, Allergan Pharmaceuticals, Dublin, Ireland [72]	1–7.5/0.1–0.3	4.3
Rabbit [67,70]	7.9 [70]	*Onabotulinumtoxin A*; Botox®, Allergan Inc., Irvine, CA, USA [67,70]	10/0.25	1.2
Human [8,71]	20.14 [83]	*Onabotulinumtoxin A*; Neuronox®, Medytox Inc., Seoul, Korea [8] *Abobotulinum A*; Dysport®, Ipsen Biopharm Ltd, Wrexham, UK [71]	25/0.5 [8] 120/0.6 [71]	3.6
